# A Sulfonated Polyimide/Nafion Blend Membrane with High Proton Selectivity and Remarkable Stability for Vanadium Redox Flow Battery

**DOI:** 10.3390/membranes11120946

**Published:** 2021-11-29

**Authors:** Jinchao Li, Jun Liu, Wenjie Xu, Jun Long, Wenheng Huang, Zhen He, Suqin Liu, Yaping Zhang

**Affiliations:** 1 State Key Laboratory of Environment-Friendly Energy Materials, School of Materials Science and Engineering, Southwest University of Science and Technology, Mianyang 621010, China; lijinchao@swust.edu.cn (J.L.); liujun9526@163.com (J.L.); xuwenjie996@163.com (W.X.); longjun0824@163.com (J.L.); sussica@126.com (W.H.); 2 College of Chemistry and Chemical Engineering, Central South University, Changsha 410083, China; zhenhe@csu.edu.cn

**Keywords:** sulfonated polyimide, blend membrane, vanadium redox flow battery, chemical stability

## Abstract

A sulfonated polyimide (SPI)/Nafion blend membrane composed of a designed and synthesized SPI polymer and the commercial Nafion polymer is prepared by a facile solution casting method for vanadium redox flow battery (VRFB). Similar molecular structures of both SPI and Nafion provide good compatibility and complementarity of the blend membrane. ATR-FTIR, ^1^H-NMR, AFM, and SEM are used to gain insights on the chemical structure and morphology of the blend membrane. Fortunately, the chemical stability of the SPI/Nafion blend membrane is effectively improved compared with reported SPI-based membranes for VRFB applications. In cycling charge-discharge tests, the VRFB with the as-prepared SPI/Nafion blend membrane shows excellent battery efficiencies and operational stability. Above results indicate that the SPI/Nafion blend membrane is a promising candidate for VRFB application. This work opens up a new possibility for fabricating high-performance SPI-based blend membrane by introduction of a polymer with a similar molecular structure and special functional groups into the SPI polymer.

## 1. Introduction

Clean and renewable energy sources such as wind and sunlight have gained much attention due to their low environmental impact, abundant reserve, and extensive distribution [1]. However, these renewable energy sources are unpredictable and fluctuant with time and season. Therefore, durable and reliable large-scale energy storage technologies are urgently needed to store the energy generated from these renewable resources and smoothly output the stored energy (usually as electricity) on demand [2,3]. The vanadium redox flow battery (VRFB) is considered as one of the most promising candidates for large-scale energy storage due to its flexible design, large storage capacity, long cycle life, high safety, fast response time, and environmental friendliness [4,5]. In the past few decades, exciting progresses have been achieved in VRFB technologies [6,7,8].

As one of the key components of VRFB, the proton conductive membrane (PCM) is used to separate the positive and negative electrolytes and allows the transport of protons to complete the circuit. An ideal PCM should possess a low vanadium ion permeability, high proton conductivity, excellent chemical stability, and low cost [9,10]. Currently, the most widely used PCMs are perfluorosulfonic acid membranes, such as Nafion series membranes from DuPont Co., USA, which possess excellent proton conductivities and chemical stability [11,12,13]. However, Nafion membranes are limited in commercial application of VRFBs due to the following issues. First, serious vanadium ion permeability results in fast self-discharge of VRFB. Second, poor proton selectivity lowers the battery efficiencies. Third, the manufacturing cost (i.e., 500–700 dollar m^−2^) is high [14,15]. Thus, a series of sulfonated aromatic polymer membranes, such as sulfonated poly(fluorenyl ether ketone) (SPFEK), sulfonated poly(ether ether ketone) (SPEEK), sulfonated polyimide (SPI), sulfonated poly(arylene ether ketone) (SPAEK), and sulfonated poly(phenylene sulfide sulfone) (SPSS) membranes have been investigated intensively as alternatives to Nafion membranes in VRFBs [16,17,18,19,20]. Among these sulfonated aromatic polymer membranes, the SPI membranes have shown lower vanadium ion permeability, better proton selectivity and thermal stability, excellent VRFB performance and lower cost compared to Nafion membranes [18,21,22]. However, the poor chemical stability of these pure SPI membranes hinders their further commercial application in VRFBs [23].

In our previous work, a side chain-type fluorinated SPI membrane with trifluoromethyl (-CF_3_) groups and flexible sulfoalkyl pendants showed excellent performance in VRFB application [24]. However, the chemical stability of this side chain-type fluorinated SPI membrane is lower than that of commercial Nafion 115 membrane. Fortunately, some inorganic or organic materials could be introduced into sulfonated aromatic polymers by blending to prepare blend membranes with high chemical stability and excellent VRFB performance [25,26,27,28,29]. Therefore, a novel SPI/Nafion blend membrane is fabricated by using Nafion polymer as a reinforcer to further optimize the physico-chemical properties of the side chain-type fluorinated SPI membrane in this work due to the following reasons [9,30]. First, the molecular structure of Nafion polymer (i.e., flexible perfluorinated side chains terminated with sulfonate groups and tetrafluoroethylene backbones) is similar to that of the side chain-type fluorinated SPI polymer (i.e., flexible sulfoalkyl pendants and aromatic backbones) as shown in Figure S1, which can provide good compatibility and is beneficial to the formation of SPI/Nafion blend membrane. In addition, Nafion polymer possesses good chemical stability, which is beneficial to the stability of SPI/Nafion blend membrane. Moreover, the abundant electron-withdrawing groups in Nafion polymer can effectively decrease the overall electron density of SPI/Nafion blend membrane, which could improve its resistance toward the oxidative species with positive charges in the VRFB electrolytes [24]. To the best of our knowledge, the SPI/Nafion blend membrane is prepared by using Nafion polymer as a filler and applied in VRFB for the first time. In addition, the solubility behavior of SPI polymer and the membrane-forming property of SPI/Nafion mixed solution are investigated. The chemical structure of SPI/Nafion blend membrane is characterized and compared with SPI membrane. The comparison of the physico-chemical properties of SPI/Nafion blend membrane and pure SPI membrane is listed in Table S1. The morphologies and physico-chemical properties of both SPI/Nafion and Nafion 115 membranes and their VRFB single cell performance are studied and compared (Table S2). The SPI/Nafion blend membrane shows a significantly lower vanadium ion permeability, higher proton selectivity, excellent thermal stability, and better VRFB performance than Nafion 115 membrane. At the same time, the SPI/Nafion blend membrane also reveals a superior ex situ chemical stability compared to the SPI-based membranes reported for VRFBs.

## 2. Materials and Methods

The SPI polymer containing -CF_3_ groups and flexible sulfoalkyl pendants was synthesized from 1,4,5,8-naphthalenetetra-carboxylic dianhydride (NTDA, Beijing Multi. Tech., Beijing, China), 4,4′-diamino-biphenyl 2,2′-disulphonic acid (BDSA, Energy Chemical. Co., Shanghai, China), 2,2-Bis[4-(4-aminophenoxy) phenyl]hexafluoropropane (HFBAPP, Changzhou Sunlight Pharmaceutical Co., Ltd., Changzhou, China), 2,2-Bis(3-amino-4-hydroxyphenyl)hexafluoropropane (APAF, Changzhou Sunlight Pharmaceutical Co., Ltd., Changzhou, China), and 1,3-propane sultone (Shanghai Aladdin Industry Co., Shanghai, China) by high-temperature polycondensation and grafting reactions according to our previously reported method [24]. Nafion 115 membrane (DuPont Co., Wilmington, DE, USA) with a thickness of 120 μm was chosen as a reference of Nafion series membranes because of its proper thickness. A membrane thinner than 100 μm, such as Nafion 211 (25 μm), Nafion 212 (50 μm), and Nafion 1135 (88 μm), would cause serious vanadium ion permeability, whereas a too thick membrane such as Nafion 117 (175 μm) with a high area resistance is not beneficial for the voltage efficiency of VRFBs [31]. Similarly, Nafion 115 membrane was also chosen as a reference by Teng, X.G. et al. [32], Ding, L.M. et al. [33], and Chen, D.J. et al. [34]. The pretreatment process of Nafion 115 membrane is shown in Section S1 in the SI.

The SPI/Nafion blend membrane was prepared using a facile solution casting method. The SPI polymer (2.0 g) was dissolved in 50.0 mL of *m*-cresol to form a 4% *w*/*v* solution. Subsequently, 4.0 g of Nafion polymer solution (5 wt.%) was slowly added, and then the mixture was stirred for 24 h to obtain a homogeneous solution. Finally, this homogeneous solution was cast onto a clean and dry glass plate and kept at 60 °C for 48 h to fully evaporate the solvent and form the SPI/Nafion blend membrane. The SPI/Nafion blend membrane was peeled off the glass plate and then immersed in 1.0 mol L^−1^ H_2_SO_4_ at room temperature for 24 h to complete the proton exchange process. Then, the SPI/Nafion blend membrane was put into deionized water (DI water) for 24 h to completely wash out the residual H_2_SO_4_ and organic solvent and stored in DI water for further use.

It is worth mentioning that the pure SPI membrane was mainly prepared by using *m*-cresol as the solvent in previous research [18,21,22,23,26,27]. Hereinto, we mainly investigated the SPI/Nafion blend membrane by using *m*-cresol as the membrane-casting solvent for better comparison in this work. However, additional solvents were also used to dissolve the SPI polymer and their effects on the membrane-forming property of SPI/Nafion mixed solution were briefly studied. In addition, the characterizations of the membranes, including ATR-FTIR, ^1^H-NMR, AFM, SEM-EDS, TGA, DMA, physico-chemical properties and VRFB single cell tests, are described in Section S2 in the SI.

## 3. Results and Discussion

### 3.1. Solubility Behavior, Membrane-Forming Property, and Chemical Structure

**Figure 1:**
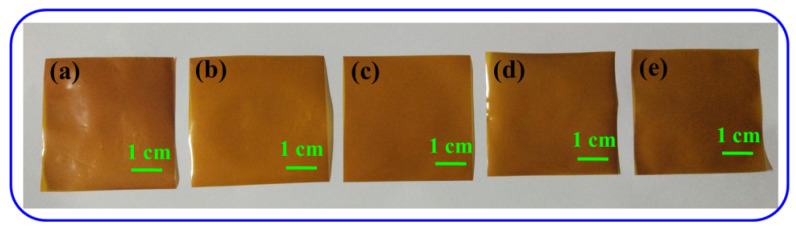
Optical photos of the SPI/Nafion blend membranes with a size of 5.0 cm × 5.0 cm fabricated using (**a**) *m*-cresol, (**b**) DMSO, (**c**) DMF, (**d**) NMP, and (**e**) DMAc as the membrane-casting solvent.

The SPI polymer needs to be dissolved in an organic solvent before addition of Nafion solution during preparation of SPI/Nafion blend membrane. Therefore, the solubility behavior of SPI polymer was investigated by the following method, and the result is presented in Table 1. About 2.0 g of the SPI polymer and 50.0 mL of a selected solvent were added into a 100 mL beaker and heated at 40 °C for 48 h under mechanical stirring. It is observed that the SPI polymer can be dissolved in various common organic solvents such as *m*-cresol, dimethyl sulfoxide (DMSO), *N*,*N*-dimethylformamide (DMF), *N*-methyl-2-pyrrolidone (NMP), and *N*,*N*-dimethylacetamide (DMAc) to form clear and homogeneous solutions. This might be due to the combined effects of ether linkages (-O-) (forming a different intrasegmental configuration) and -CF_3_ groups (disrupting the regularity of the molecular chains) [35]. Besides, the optical photos of SPI/Nafion blend membranes are shown in Figure 1. The light-brown SPI/Nafion blend membranes are homogeneous, dense and smooth, suggesting that the corresponding SPI/Nafion mixed solution has excellent compatibility and membrane-forming property.

**Figure 2:**
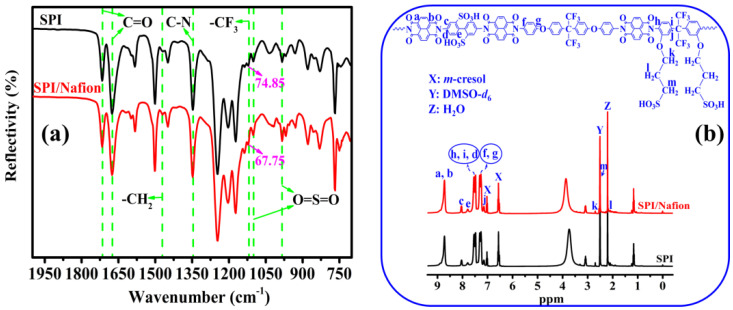
(**a**) ATR−FTIR and (**b**) ^1^H-NMR spectra of the SPI (black curves) and SPI/Nafion (red curves) membranes.

The chemical structures of SPI/Nafion and SPI membranes were characterized by ATR-FTIR and ^1^H-NMR, and the results are presented in Figure 2. The strong absorption bands around 1716.36 and 1675.87 cm^−1^ are related to the symmetric and asymmetric stretching vibrations of the carbonyl (C=O) groups in naphthalimide rings [21]. The C-N asymmetric stretching vibration of imide rings and the vibration of methylene (-CH_2_) groups appear at 1348.02 and 1469.52 cm^−1^, respectively. The absorption bands at 1101.17 and 985.87 cm^−1^ could be assigned to the stretching vibrations of sulfonic acid (-SO_3_H) groups [18]. The absorption peak at 1120.46 cm^−1^ corresponds to the C-F stretching [24]. Herein, the reflectivity of the peak of the SPI/Nafion blend membrane (67.75%) is lower than that of the SPI membrane (74.85%), suggesting that the number of C-F bonds in the SPI/Nafion blend membrane is significantly increased by incorporating Nafion polymer into the SPI membrane. Besides, the ^1^H-NMR spectrum of the SPI/Nafion blend membrane is almost the same as that of the SPI membrane (Figure 2b), because the Nafion polymer with a perfluorinated structure has no ^1^H-NMR detectable proton. The ^1^H-NMR spectral signals can be reasonably assigned to different protons in the repeated units of the SPI polymer. The characteristic peak at 8.72 ppm is attributed to the protons (H_a_ and H_b_) in the naphthalene rings of the SPI polymer. In addition, the signals between 2.0 and 2.8 ppm are assigned to the alkyl hydrogens (H_k_, H_m_, and H_l_) of the flexible sulfoalkyl pendants. The appearance of peaks between 7.1 and 8.2 ppm could be assigned to the hydrogen atoms (H_c_, H_d_, H_e_, H_f_, H_g_, H_h_, H_i_, and H_j_) on the benzene rings of BDSA, HFBAPP, and APAF. The ATR-FTIR and ^1^H-NMR spectra results show that the chemical structure of the SPI/Nafion blend membrane is similar to that of the SPI membrane although Nafion polymer has been incorporated. In addition, the ATR-FTIR and ^1^H-NMR spectra of the SPI/Nafion blend membranes prepared using DMSO, NMP, DMF, and DMAc as the membrane-casting solvents were also investigated (Figures S2 and S3). All these SPI/Nafion blend membranes have almost the same ATR-FTIR and ^1^H-NMR spectra, meaning that the membrane-casting solvent has no obvious effect on the chemical structure of the SPI/Nafion blend membrane.

### 3.2. Membrane Morphology

**Figure 3:**
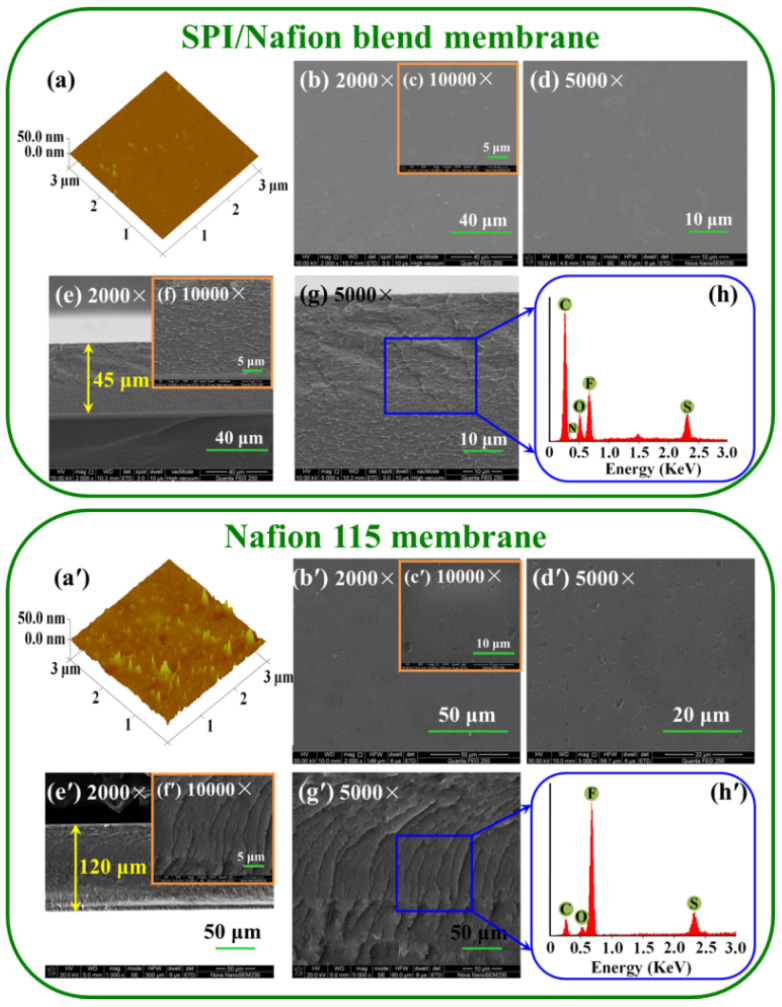
AFM images of (**a**) SPI/Nafion and (**a**′) Nafion 115 membranes. SEM surface images of (**b**–**d**) SPI/Nafion and (**b**′−**d**′) Nafion 115 membranes. SEM cross-sectional images of (**e**–**g**) SPI/Nafion and (**e**′–**g**′) Nafion 115 membranes. The EDS analysis of the cross-section of (**h**) SPI/Nafion and (**h**′) Nafion 115 membranes.

The surface and cross-sectional morphologies of both SPI/Nafion and Nafion 115 membranes were investigated using AFM and SEM (as shown in Figure 3). The peak-valley difference of SPI/Nafion blend membrane is significantly smaller than that of the Nafion 115 membrane. The surface roughness parameter of the SPI/Nafion blend membrane (Ra = 0.418 nm) is also much lower than that of Nafion 115 membrane (Ra = 1.83 nm). The surface area differences of the SPI/Nafion and Nafion 115 membranes are 0.110% and 0.965%, respectively. Besides, the SEM images with different magnifications show that the surface of SPI/Nafion blend membrane is dense with no pinholes or cracks (Figure 3b–d), whereas the surface of Nafion 115 membrane is covered with some dents (Figure 3b′–d′). These results show that the surface of SPI/Nafion blend membrane is more uniform and smoother than that of Nafion 115 membrane. The SPI polymer has excellent solubility and membrane-forming property. Thus, the SPI membrane can obtain smooth and uniform surface morphology. Similar results were also reported by Long, J. et al. and Yang, P. et al. [36,37]. In this work, the blending ratio of Nafion polymer is very low (only 10 wt.%), so the surface morphology of SPI/Nafion blend membrane can maintain smooth and uniform as pure SPI membrane. In comparison, Nafion membrane exhibits a relatively rougher surface morphology as was observed in a previous report [38]. Moreover, the cross-sections of the SPI/Nafion (Figure 3e–g) and Nafion 115 membranes (Figure 3e′–g′) are dense at different magnifications. The elements in the SPI/Nafion and Nafion 115 membranes were also studied by EDS (Figure 3h,h′). The content of F in the SPI/Nafion blend membrane is much lower than that in Nafion 115 membrane. This is because only a small quantity of Nafion polymer is introduced into the SPI/Nafion blend membrane.

### 3.3. Rheological Property and Thermal Stability

**Figure 4:**
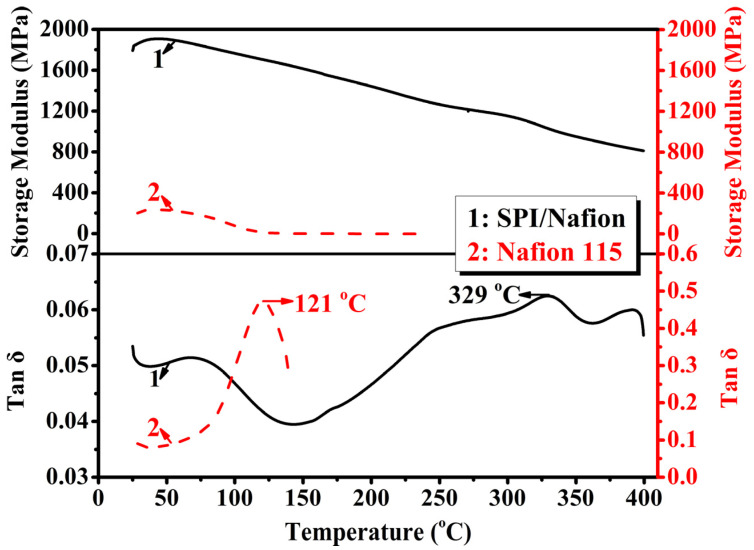
The storage modulus and tan δ curves changed with temperature under a nitrogen atmosphere, and *T*_g_ values of SPI/Nafion and Nafion 115 membranes.

High mechanical strength could provide long-term durability for the PCM during the VRFB operation [39]. Therefore, the rheological properties of SPI/Nafion and Nafion 115 membranes were investigated by DMA, and the plots are shown in Figure 4. Storage modulus is a measure of the stiffness of membrane at a given temperature [40]. The SPI/Nafion blend membrane has a significantly higher storage modulus in the temperature range of 25 to 400 °C compared to Nafion 115 membrane. The Nafion 115 membrane becomes rubbery at 242 °C, whereas the SPI/Nafion blend membrane retains stiffness even up to 400 °C (with a storage modulus of about 810.6 MPa). The maximum tan δ value for membrane is taken to represent the glass transition temperature (*T*_g_). The *T*_g_ can be expressed as the temperature of the polymer starting the molecular Brownian motion [41]. The SPI/Nafion blend membrane has a higher *T*_g_ (329 °C) compared to the Nafion 115 membrane (121 °C), suggesting that the SPI/Nafion blend membrane could retain dimensional stability up to 329 °C. The SPI/Nafion blend membrane has a significantly higher storage modulus and *T*_g_ compared to Nafion 115 membrane. This is possibly because that SPI polymer with solid aromatic backbone structure occupies the main component of SPI/Nafion blend membrane. The introduction of 10 wt.% Nafion polymer does not affect the rheological properties of SPI membrane. The result is also proved through the comparison of mechanical properties (tensile strength, Young’s modulus and elongation at break) between SPI and SPI/Nafion blend membranes.

In addition, the TGA curves of SPI/Nafion blend membranes fabricated using different membrane-casting solvents (i.e., *m*-cresol, DMSO, DMF, NMP or DMAc), Nafion 115 membrane, and pure SPI membrane are presented in Figure S4. The thermal stability of SPI/Nafion blend membranes is slightly lower than pure SPI membrane, resulting from the incorporation of Nafion polymer with poor thermal stability into SPI membrane. The Nafion polymer with poor thermal stability could be attributed to its special perfluorosulfonic acid molecular structure. The first step for decomposition of Nafion polymer can be attributed to the flexible perfluorinated side chains terminated with sulfonic acid groups (-O-CF_2_-CF_2_-SO_3_H), while the second weight loss step can be attributed to the degradation of main-chain (-CF_2_-CF_2_-) units. The O-CF_2_-CF_2_- unit has destabilizing effect on thermal stability of the sulfonic acid group [42]. Besides, the Nafion polymer has low glass transition temperature, resulting in that its molecular chains are prone to Brownian motion [41]. However, the thermal stability of these SPI/Nafion blend membranes is superior to that of Nafion 115 membrane. The SPI/Nafion blend membrane fabricated using *m*-cresol as the solvent has the highest weight retention among all the tested blend membranes at 800 °C, suggesting that it has the best thermal stability among these blend membranes. This is possibly because that *m*-cresol has the high boiling point, and the evaporation time (48 h) of *m*-cresol solvent is longer than that of other solvents including DMSO, DMF, NMP, and DMAc in the SPI/Nafion blend membrane-forming process. Long evaporation time of *m*-cresol could be beneficial to the rearrangement and crystallization of macromolecules, as a result, the SPI/Nafion blend membrane using *m*-cresol solvent can obtain a slightly higher thermal stability [43]. These results suggest that the as-prepared SPI/Nafion blend membrane is thermally stable enough for VRFB application [44].

### 3.4. Water Uptake, Swelling Ratio, Contact Angle, and Mechanical Property

The water uptake (*WU*) and swelling ratio (*SR*) of SPI/Nafion and Nafion 115 membranes are illustrated in Table 2. The *WU*s of SPI/Nafion blend membrane are 16.92% at 20 °C and 19.31% at 40 °C, which are lower compared with that of SPI membrane (17.78% at 20 °C and 22.22% at 40 °C) [24]. This is due to the existence of the strongly hydrophobic Nafion polymer with a poly(tetrafluoroethylene) main-chain structure in the blend membrane, which is more effective in repelling water molecules [9]. A low *SR* is beneficial to the dimensional stability of the membrane. Thus, the through-plane (based on the change of thickness) and in-plane (based on the change of length) *SR*s were measured at 20 and 40 °C separately. The through-plane *SR* (*SR*_Δt_) of SPI/Nafion blend membrane is higher than the in-plane *SR* (*SR*_Δl_) at both 20 and 40 °C. The SPI/Nafion blend membrane has a lower *SR*_Δt_ (13.52% at 20 °C and 17.24% at 40 °C) than the SPI membrane (14.51% at 20 °C and 17.72% at 40 °C) [24]. This could be attributed to the restrained movement of the molecular chains arising from the enhanced entanglement of the flexible side chains between the SPI and Nafion polymers [45].

In addition, the contact angles of the SPI/Nafion and Nafion 115 membranes by using DI water, 3.0 mol L^−1^ H_2_SO_4_ solution, and 1.5 mol L^−1^ VO^2+^ + 3.0 mol L^−1^ H_2_SO_4_ solution (i.e., a VRFB electrolyte) as probes are shown in Figure S5. The contact angles of SPI/Nafion and Nafion 115 membranes have the same order, 3.0 mol L^−1^ H_2_SO_4_ > 1.5 mol L^−1^ VO^2+^ + 3.0 mol L^−1^ H_2_SO_4_ > DI water. The water contact angle of SPI/Nafion blend membrane (87.8°) is larger than that of SPI membrane (83.5°), which agrees well with their *WU* results [24]. As expected, since Nafion 115 membrane has the lowest *WU* (15.28% at 20 °C and 17.68% at 40 °C), *SR*_Δt_ (11.96% at 20 °C and 16.10% at 40 °C), and *SR*_Δl_ (2.35% at 20 °C and 3.49% at 40 °C), it has the largest water contact angle (101.5°) on account of its hydrophobic poly(tetrafluoroethylene) main-chain structure [21].

### 3.5. Ion Exchange Capacity, Proton Conductivity, Vanadium Ion Permeability and Proton Selectivity

The ion exchange capacities (*IEC*s) of SPI/Nafion and Nafion 115 membranes are shown in Table 2. The *IEC* is an important performance index of PCM [46]. The *IEC* of SPI/Nafion blend membrane (1.61 meq g^−1^) is much larger than that of Nafion 115 membrane (0.74 meq g^−1^), meaning that the SPI/Nafion blend membrane can obtain sufficient ionic exchange groups [22]. The SPI/Nafion blend membrane with an excellent *IEC* can provide a satisfactory proton conductivity (*σ*). The *σ* values of SPI/Nafion and Nafion 115 membranes are listed in Table 2. The area resistance (*AR*) of SPI/Nafion blend membrane (0.22 Ω cm^2^) is slightly higher than that of Nafion 115 membrane (0.20 Ω cm^2^). This could be attributed to the structure difference between SPI (with a robust aromatic backbone) and Nafion (with a flexible aliphatic backbone). This result means that the VRFB assembled with SPI/Nafion blend membrane will probably have a higher ohmic loss in the charge-discharge process [47]. The *σ* of SPI/Nafion blend membrane (2.05 × 10^−2^ S cm^−1^) is lower than that of Nafion 115 membrane (6.00 × 10^−2^ S cm^−1^) because the SPI/Nafion blend membrane has a slightly higher *AR* and thinner thickness than Nafion 115 membrane. Besides, the Nafion 115 membrane has a unique micro-phase separation that is beneficial for the *σ* [21]. However, the *σ* of SPI/Nafion blend membrane is twice as large as the commercially acceptable value of 0.01 S cm^−1^, meaning that the as-prepared blend membrane is applicable for VRFB [27].

**Figure 5:**
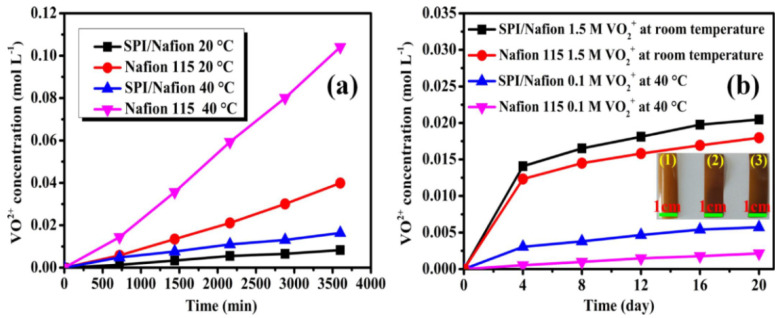
(**a**) Variation of the concentration of VO^2+^ ions across SPI/Nafion and Nafion 115 membranes as a function of time at 20 and 40 °C. (**b**) Ex-situ chemical stability study of SPI/Nafion and Nafion 115 membranes. The inset in (**b**) are the optical photos of SPI/Nafion blend membrane with a size of 3.0 cm × 1.0 cm: (1) fresh membrane; (2) soaked in 0.1 mol L^−1^ VO_2_^+^ + 3.0 mol L^−1^ H_2_SO_4_ solution at 40 °C for 20 days; (3) soaked in 1.5 mol L^−1^ VO_2_^+^ + 3.0 mol L^−1^ H_2_SO_4_ solution at room temperature for 20 days.

The vanadium ion permeability (*P*) is an important performance parameter to assess the ability of membrane to prevent the crossover of vanadium ions for VRFB application [48]. Low *P* of the membrane can effectively improve the coulombic efficiency of VRFB. Thus, the rate of VO^2+^ permeation was measured in a diffusion cell (Scheme S1), and a linear relationship between the permeated VO^2+^ ion concentration and time is shown in Figure 5a. Meanwhile, the *P*s of SPI/Nafion and Nafion 115 membranes are listed in Table 2. The *P*s of SPI/Nafion blend membrane are 1.25 × 10^−7^ cm^2^ min^−1^ at 20 °C and 2.23 × 10^−7^ cm^2^ min^−1^ at 40 °C respectively, which are much lower compared to those of Nafion 115 (i.e., 13.59 × 10^−7^ cm^2^ min^−1^ at 20 °C and 35.97 × 10^−7^ cm^2^ min^−1^ at 40 °C individually). These results can be attributed to two main factors. On one hand, the VO^2+^ ion transport channels are narrowed and branched in the SPI polymer. On the other hand, the chain packing density of SPI/Nafion blend membrane is increased due to the entanglement between the flexible side chains of SPI and Nafion polymers [24]. Therefore, the SPI/Nafion blend membrane has a strong ability to suppress the crossover of vanadium ions between positive and negative electrolytes in the operational process of VRFB.

A PCM is required to possess both high *σ* and low *P* [48]. Generally, the proton selectivity (*PS*) can evaluate the combined effect of *σ* and *P* for a PCM. The SPI/Nafion blend membrane exhibits an excellent *PS* (1.64 × 10^5^ S min cm^−3^), which is around 3.3 times higher than that of Nafion 115 membrane (0.44 × 10^5^ S min cm^−3^). The SPI/Nafion blend membrane with a high *PS* is expected to display outstanding performance in VRFB.

The SPI/Nafion blend membrane exhibits a higher tensile strength (68.62 MPa) and Young’s modulus (1.09 GPa) compared to SPI membrane (50.97 MPa and 0.81 GPa) and Nafion 115 membrane (12.79 MPa and 0.06 GPa) [24]. The elongation at break of SPI/Nafion blend membrane (100.00%) and SPI membrane (58.01%) is lower compared with that of Nafion 115 membrane (223.17%), because Nafion 115 membrane has flexible aliphatic main chains [18,24]. However, the elongation at break of SPI/Nafion blend membrane is superior to that of SPI membrane, which is possibly due to the addition of Nafion polymer with aliphatic main chains. Besides, the Nafion polymer with flexible side chains can indeed improve the entanglement of all polymer chains [22]. This result shows that the elongation at break of SPI/Nafion blend membrane could be effectively enhanced by the incorporation of Nafion polymer, which provides an effective method for improving the strain of sulfonated aromatic polymer membrane.

### 3.6. Ex-Situ Chemical Stability

The chemical stability of PCMs directly affects the lifetime and performance of VRFBs. The chemical stability of membrane was measured through two ex situ test methods (Method 1: soaking the membrane in a 0.1 mol L^−1^ VO_2_^+^ + 3.0 mol L^−1^ H_2_SO_4_ solution at 40 °C; Method 2: soaking the membrane in a 1.5 mol L^−1^ VO_2_^+^ + 3.0 mol L^−1^ H_2_SO_4_ solution at room temperature). The highly oxidizing VO_2_^+^ ions in the solution could oxidize the membrane and be reduced to VO^2+^ ions [23]. Therefore, the concentration of VO^2+^ ions generated during the experiment is illustrated in Figure 5b. The concentrations of VO^2+^ ions in the soaking solutions gradually increase as the immersing time increases in both Method 1 and 2. The comparison of ex−situ chemical stability between SPI/Nafion blend membrane and other SPI-based membranes are presented in Table S3. The chemical stability of SPI/Nafion blend membrane is superior to reported SPI-based membranes under similar ex-situ test conditions [18,21,22,24,25,49,50,51,52,53,54]. This is probably due to two reasons. First, the water uptake of SPI/Nafion blend membrane is lower than that of pure SPI membrane, which is beneficial for protecting the imide rings in SPI polymer from being attacked by the hydrolytic species like H^+^ ions [23,55]. Moreover, the abundant electron-withdrawing groups in Nafion polymer can effectively decrease the overall electron density of SPI/Nafion blend membrane, which could improve its resistance toward the oxidative species with positive charges like VO_2_^+^ ions [9,56]. The optical photos of SPI/Nafion blend membranes soaked in 0.1 mol L^−1^ VO_2_^+^ + 3.0 mol L^−1^ H_2_SO_4_ solution at 40 °C and 1.5 mol L^−1^ VO_2_^+^ + 3.0 mol L^−1^ H_2_SO_4_ solution at room temperature after 20 days are also shown as the insets in Figure 5b. The SPI/Nafion blend membrane after the test is intact and almost has no change compared to the fresh one, meaning that the as-prepared SPI/Nafion blend membrane has excellent chemical stability. The main purpose of this work is to improve the chemical stability of SPI membrane. The chemical stability of SPI/Nafion blend membrane could be further increased if the blending ratio of Nafion polymer exceeds 10 wt.%. However, the cost of SPI/Nafion blend membrane is also increased with the increase of the content of Nafion polymer. Therefore, taking cost performance into account, 10 wt.% of Nafion polymer is determined to be an optimum ratio for preparation of SPI/Nafion blend membrane in this work.

### 3.7. Battery Performance

**Figure 6:**
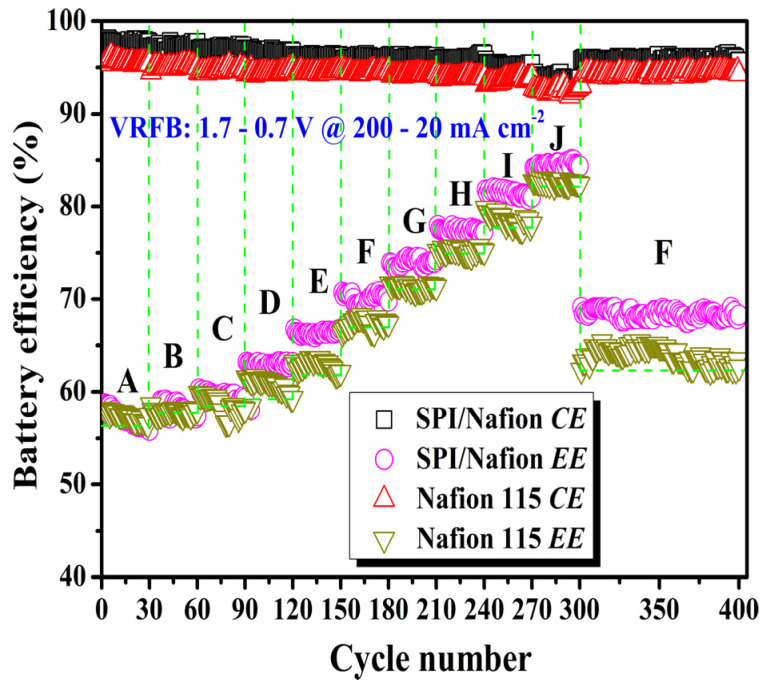
Comparison of the battery performance between SPI/Nafion and Nafion 115 membranes. (A = 200 mA cm^−2^; B = 180 mA cm^−2^; C = 160 mA cm^−2^; D = 140 mA cm^−2^; E = 120 mA cm^−2^; F = 100 mA cm^−2^; G = 80 mA cm^−2^; H = 60 mA cm^−2^; I = 40 mA cm^−2^; J = 20 mA cm^−2^).

A VRFB single cell is schematically shown in Scheme S2, in which the battery performance of SPI/Nafion and Nafion 115 membranes is evaluated. The charge-discharge cycle tests were first carried out for 300 cycles at different current densities (in the range of 200 to 20 mA cm^−2^). The coulombic efficiencies (*CE*s), voltage efficiencies (*VE*s), and energy efficiencies (*EE*s) are also obtained by calculating the average values of the efficiencies of 30 cycles at each current density. All results are illustrated in Figure 6 and Figure S6. The *CE*s of SPI/Nafion blend membrane (97.77–93.88%) are higher than those of Nafion 115 membrane (95.59–92.53%) at all tested current densities. The *CE* of the VRFB increases with the increase of current density because the charge-discharge time is shortened at higher current densities, resulting in less vanadium ion permeation [36]. The *VE* of VRFB increases as the current density decreases from 200 to 20 mA cm^−2^, which is due to the lower ohmic polarization at lower current densities [37]. The *VE*s of SPI/Nafion blend membrane is higher than those of Nafion 115 membrane from 140 to 20 mA cm^−2^. This phenomenon could be attributed to two factors as follows: (i) The SPI/Nafion blend membrane keeps a better balance between vanadium ion permeability and proton conductivity. Accordingly, its rising speed of *VE* is obviously faster compared with Nafion 115 membrane while the current density decreases. (ii) The SPI/Nafion blend membrane shows no obvious ohmic loss when the current density is below 140 mA cm^−2^. However, the *VE*s of SPI/Nafion blend membrane is lower than that of Nafion 115 membrane at high current densities (e.g., 200, 180, and 160 mA cm^−2^), which could be attributed to the higher ohmic loss arising from the higher *AR* and lower *σ* of SPI/Nafion blend membrane [47]. As an electrical energy storage system, the *EE* is an important indicator of energy loss during the charge-discharge process [51]. At the current density of 200 mA cm^−2^, the *EE* of SPI/Nafion blend membrane is slightly lower than that of Nafion 115 membrane. This is possibly because the SPI/Nafion blend membrane has a higher *AR* and lower *σ* compared to Nafion 115 membrane [21]. However, the *EE*s of SPI/Nafion blend membrane are higher than those of Nafion 115 membrane at current densities changed from 180 to 20 mA cm^−2^ due to the excellent *PS* of SPI/Nafion blend membrane as discussed above (Table 2). In general, the battery performance of as-prepared SPI/Nafion blend membrane is better than that of Nafion 115 membrane. More 100-time cycling tests of VRFBs with these two membranes were conducted continuously at 100 mA cm^−2^ after the first 300-time cycling test. The *CE* and *EE* can almost attain the same values as those in the 151 to180 cycles (at 100 mA cm^−2^), and the *CE* and *EE* do not significantly decline, suggesting that the prepared SPI/Nafion blend membrane has excellent chemical/electrochemical stability to survive the VRFB environment. The discharge capacity retention results of SPI/Nafion blend membrane and Nafion 115 membrane at another 100-time cycling VRFB tests at 100 mA cm^−2^ after the first 300-time cycling test are shown in Figure S7. The discharge capacity retentions of SPI/Nafion blend membrane and Nafion 115 membrane are 77.74% and 64.01% at the 100th cycle respectively. Besides, the discharge capacity retention of SPI/Nafion blend membrane is obviously higher than Nafion 115 membrane for each cycle, further confirming that the SPI/Nafion blend membrane has solid vanadium resistance. Besides, the 500-time VRFB charge-discharge cycling test was also performed at 100 mA cm^−2^, and the result is presented in Figure S8. The SPI/Nafion blend membrane shows excellent cycling charge-discharge performance, verifying that it can endure the long-term application of VRFB. These results mean that the blending strategy has a significant prospect for fabricating high-performance SPI-based blend membrane.

**Figure 7:**
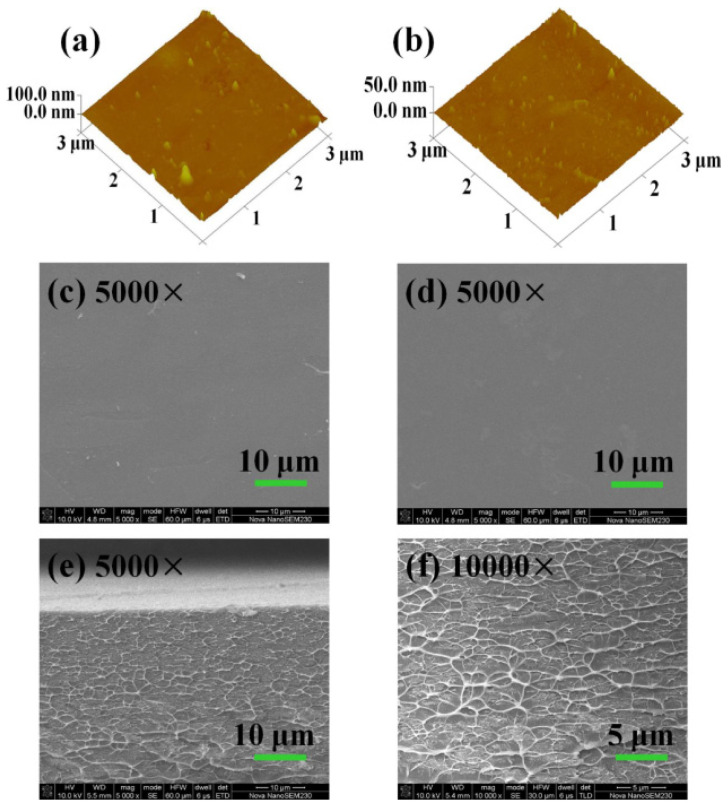
AFM images of SPI/Nafion blend membrane after the 400-time VRFB cycling test: (**a**) surface facing positive electrode and (**b**) surface facing negative electrode. SEM images of SPI/Nafion blend membrane after the 400-time VRFB cycling test: (**c**) surface facing positive electrode, (**d**) surface facing negative electrode, and (**e**,**f**) cross-section.

The surface and cross-sectional morphologies of SPI/Nafion blend membrane after a 400-time cycling charge-discharge test were studied by using AFM and SEM. As shown in Figure 7a,b, the surface roughness parameters and surface area differences of SPI/Nafion blend membrane facing the positive (1.60 nm and 0.807%) and negative (0.950 nm and 0.764%) electrodes are higher than those of fresh SPI/Nafion blend membrane (0.418 nm and 0.110%). This is probably due to the squeeze of membrane by two pieces of graphite felt electrodes during the long-term cycling test. However, the roughness of SPI/Nafion blend membrane facing the positive electrode is slightly higher than that facing the negative electrode, suggesting that the VO^2+^/VO_2_^+^ positive electrolyte with stronger oxidizability has more obvious negative impact on this blend membrane than the V^3+^/V^2+^ negative electrolyte. The SEM images of the surface and cross-section of used SPI/Nafion blend membrane are presented in Figure 7c–f. The surfaces facing the positive and negative electrodes and the cross-section of used SPI/Nafion blend membrane are almost the same as those of fresh blend membrane, indicating that the SPI/Nafion blend membrane has excellent chemical stability by the introduction of Nafion polymer.

**Figure 8:**
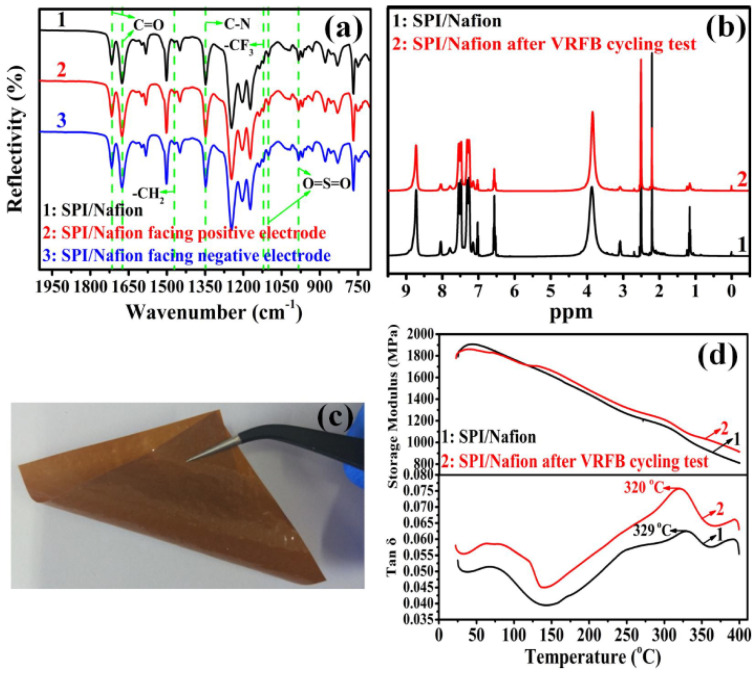
(**a**) ATR-FTIR and (**b**) ^1^H-NMR spectra of SPI/Nafion blend membrane before and after the 400-time VRFB cycling test. (**c**) The optical photo of SPI/Nafion blend membrane after the 400-time VRFB cycling test. (**d**) DMA curves of SPI/Nafion blend membrane before and after the 400-time VRFB cycling test.

The ATR-FTIR and ^1^H-NMR spectra of SPI/Nafion blend membrane after the 400-time cycling tests (Figure 8a,b) show neither new peak nor peak shifts, suggesting that the chemical environments of all the functional groups in SPI/Nafion blend membrane have not been changed. The optical photo of SPI/Nafion blend membrane after the cycling test is shown in Figure 8c. The SPI/Nafion blend membrane is intact and its color is not changed, implying that the as-prepared blend membrane is stable in the VRFB application. However, the surface of SPI/Nafion blend membrane becomes slightly corrugated after the cycling test, leading to the surface roughness increase. In addition, the DMA curves of SPI/Nafion blend membrane after the 400-time VRFB cycling test are also presented in Figure 8d. The storage modulus of SPI/Nafion blend membrane (914.5 MPa) after the 400-time VRFB cycling test is slightly higher than that of fresh SPI/Nafion blend membrane (810.6 MPa) at 400 °C. This indicates that the stiffness of SPI/Nafion blend membrane is enhanced after the cycling test, resulting from the increased cross-linking density of the blend polymer chains [24]. The storage modulus of SPI/Nafion blend membrane after the cycling test is still much higher compared to Nafion 115 membrane. More importantly, the *T*_g_ of SPI/Nafion blend membrane can attain 320 °C after the cycling test and is only slightly decreased by 9 °C compared to that of fresh one (*T*_g_ = 329 °C). The TGA curves of SPI/Nafion blend membrane before and after the 400-time VRFB cycling test are also shown in Figure S9. The weight retention of SPI/Nafion blend membrane (44.93%) after the 400-time cycling test is only slightly lower than fresh one (50.53%) and is much higher than that of Nafion 115 at 800 °C. These results demonstrate that the SPI/Nafion blend membrane has excellent rheological properties and thermal stability that is suitable for VRFB application. The morphology parameters, thermal stability, and rheological properties of SPI/Nafion after 400-time VRFB cycling test and fresh Nafion 115 membranes are listed and compared in Table S4. Based on these comparisons and analyses, we believe that the SPI/Nafion blend membrane is durable for the harsh strong acidic and oxidizing environment of VRFB.

## 4. Conclusions

In this work, a blend membrane sourced from SPI and Nafion polymers was successfully prepared for VRFB application. The AFM and SEM results indicate that the SPI/Nafion blend membrane has a dense and homogeneous morphology. The physico-chemical properties of SPI/Nafion blend membrane have shown that the addition of Nafion polymer indeed enhances the dimensional stability and mechanical properties of blend membrane. In addition, introducing Nafion polymer into a SPI membrane has been shown to be of great importance for enhancing the chemical stability of membrane. The *CE*s of SPI/Nafion blend membrane are higher than that of Nafion 115 membrane at all tested current densities due to the much lower vanadium ion permeation of blend membrane. Furthermore, the VRFB with SPI/Nafion blend membrane exhibits higher *EE*s than that with Nafion 115 membrane at current densities from 180 to 20 mA cm^−2^. The high VRFB performance of SPI/Nafion blend membrane could be attributed to its good proton selectivity and remarkable chemical and structural stability. Therefore, the SPI/Nafion blend membrane is expected to be a promising candidate membrane for VRFB. This work also provides a strategy to improve the stability of SPI-based PCMs for better application in VRFBs.

## Figures and Tables

**Table 1:** Solubility behavior of the SPI polymer in various solvents.

Solvent	*m*-Cresol	DMSO	DMF	NMP	DMAc	Anhydrous Ethanol	Methanol	Acetone	Deionized Water
Dissolving property	✓	✓	✓	✓	✓	×	×	×	×

✓: completely soluble; ×: completely insoluble.

**Table 2:** Physico-chemical properties of SPI/Nafion and Nafion 115 membranes: thickness, water uptake, through- and in-plane swelling ratios, ion exchange capacity, vanadium ion permeability, area resistance, proton conductivity, and proton selectivity.

Membrane	Thickness (μm)	*WU*/*SR*_Δt_/*SR*_Δl_ (%)	*IEC* (meq g^−1^)	*P* (×10^−7^ cm^2^ min^−1^)	*AR* (Ω cm^2^)	*σ* (×10^−2^ S cm^−1^)	*PS* (×10^5^ S min cm^−3^)
SPI/Nafion	45	16.92/13.52/2.57 (20 °C) 19.31/17.24/3.87 (40 °C)	1.61	1.25 (20 °C) 2.23 (40 °C)	0.22	2.05	1.64
Nafion 115	120	15.28/11.96/2.35 (20 °C) 17.68/16.10/3.49 (40 °C)	0.74	13.59 (20 °C) 35.97 (40 °C)	0.20	6.00	0.44

## Data Availability

Not applicable.

## Supplementary Materials

The following are available online at https://www.mdpi.com/article/10.3390/membranes11120946/s1. Scheme S1: The measurement device of vanadium ion permeability. (1) The left half-cell filled with 1.0 mol L^−1^ VOSO_4_ + 2.0 mol L^−1^ H_2_SO_4_; (2) the sample membrane; (3) the right half-cell filled with 1.0 mol L^−1^ MgSO_4_ + 2.0 mol L^−1^ H_2_SO_4_. Scheme S2: Schematic illustration of a VRFB single cell. (a) liquid inlet; (b) liquid outlet; (c) gasket; (d) copper foil; (e) graphite bipolar plate; (f) plastic frame; (g) carbon felt; and (h) membrane. The plastic frame is used to hold the carbon felt and is basically in the same plane as the carbon felt. Figure S1: The molecular structures of SPI and Nafion polymers. Figure S2: The ATR-FTIR spectra of SPI/Nafion blend membranes fabricated using *m*-cresol, DMSO, DMF, NMP, and DMAc as the membrane-casting solvents, respectively. Figure S3: The ^1^H-NMR spectra of SPI/Nafion blend membranes fabricated using *m*-cresol, DMSO, DMF, NMP, and DMAc as the membrane-casting solvents, respectively. Figure S4: TGA curves of the pure SPI membrane, the Nafion 115 membrane, and the SPI/Nafion blend membranes fabricated using different solvents including *m*-cresol, DMSO, DMF, NMP, and DMAc. Figure S5: The contact angles of (a–c) SPI/Nafion and (d–f) Nafion 115 membranes. The concentration of H_2_SO_4_ is 3.0 mol L^−1^. The electrolyte consists of 1.5 mol L^−1^ VO^2+^ and 3.0 mol L^−1^ H_2_SO_4_. Figure S6: The coulombic efficiencies (*CE*s), energy efficiencies (*EE*s), and voltage efficiencies (*VE*s) of SPI/Nafion and Nafion 115 membranes under current densities from 200 to 20 mA cm^−2^. Figure S7: The discharge capacity retentions of SPI/Nafion and Nafion 115 membranes at 100 mA cm^−2^. Figure S8: The 500-time cycling performance of SPI/Nafion blend membrane at 100 mA cm^−2^. Figure S9: TGA curves of SPI/Nafion membrane before and after 400-time VRFB cycling test. Table S1: The physico-chemical properties and VRFB performance of pure SPI and SPI/Nafion blend membranes. Table S2: Comparisons of the morphologies, physico-chemical properties, and battery performance of VRFBs assembled with SPI/Nafion and Nafion 115 membranes. The properties of SPI/Nafion membrane superior to those of Nafion 115 membrane are marked in red. Table S3: Comparison of the ex situ chemical stability of SPI/Nafion blend membrane and other SPI-based membranes. Table S4: Comparisons of the morphology parameters, thermal stability, and rheological properties between SPI/Nafion after 400-time VRFB cycling test and fresh Nafion 115 membranes. The properties of SPI/Nafion blend membrane after 400−time VRFB cycling test superior to those of Nafion 115 membrane are marked in red.
