# Supplementary material for: A Sulfonated Polyimide/Nafion Blend Membrane with High Proton Selectivity and Remarkable Stability for Vanadium Redox Flow Battery
Source: Membranes (Basel). 2021 Nov 29;11(12):946. doi: 10.3390/membranes11120946 (PMC8708936; doi:10.3390/membranes11120946)
Supplement: Supplementary file 1 [file membranes-11-00946-s001.zip › membranes-1486119 - suppl - updated.pdf]

membranes

Article

Supplementary Materials: A Sulfonated Polyimide/Nafion Blend Membrane with High Proton Selectivity and Remarkable Stability for Vanadium Redox Flow Battery

Jinchao Li [1,2], Jun Liu [1], Wenjie Xu [1], Jun Long [1], Wenheng Huang [1], Zhen He [2], Suqin Liu [2,*] and Yaping Zhang [1,*]

[1] State Key Laboratory of Environment-friendly Energy Materials, School of Materials Science and Engineering, Southwest University of Science and Technology, Mianyang, 621010, PR China; lijinchao@swust.edu.cn (J.L.); liujun9526@163.com (J.L.); xuwenjie996@163.com (W.X.); longjun0824@163.com (J.L.); sussica@126.com (W.H.)

[2] College of Chemistry and Chemical Engineering, Central South University, Changsha, 410083, PR China; zhenhe@csu.edu.cn

* Correspondence: sqliu2003@126.com (S.L.); zhangyaping@swust.edu.cn (Y.Z.); Tel.: +86-816-6089372 (Y.Z.)

1. Materials

1,4,5,8-naphthalenetetra-carboxylic dianhydride (NTDA) was purchased from Beijing Multi. Tech., China. 4,4'-diamino-biphenyl 2,2'-disulphonic acid (BDSA) was bought from Energy Chemical. Co., China. 2,2-Bis(3-amino-4-hydroxyphenyl)hexafluoropropane (APAF) and 2,2-Bis[4-(4-aminophenoxy) phenyl]hexafluoropropane (HFBAPP) were from Changzhou Sunlight Pharmaceutical Co., Ltd, China. 1,3-propane sultone and N-methyl-2-pyrrolidone (NMP) were bought from Shanghai Aladdin Industry Co., China. The 5 wt.% Nafion solution was obtained from Hunan Dovop Electric Co., Ltd, China. *m*-cresol was obtained from J&K Scientific Ltd, China. Vanadyl sulfate was purchased from Shanghai Huating Chem. Plant, China. Triethylamine, benzoic acid, dimethyl sulfoxide (DMSO), N,N-dimethylformamide (DMF), N,N-dimethylacetamide (DMAc), and other reagents were obtained from Sinopharm Chemical Reagent Co., Ltd, China. All the chemicals were used as received without any further purification. Nafion 115 membrane was supplied from DuPont Co., USA, which was pretreated according to the previously reported method [1]. Initially, Nafion 115 membrane was pretreated in 3.0 wt% H_2O_2 solution at 70 °C for 0.5 h and then taken out and washed with deionized (DI) water. Afterwards, Nafion 115 membrane was immersed in 0.05 mol L^{-1} H_2SO_4 solution at 70 °C for 0.5 h and then taken out and washed with DI water. Finally, the pretreated Nafion 115 membrane with a thickness of 120 μm was stored in DI water for further use.

2. Characterization Methods

2.1. ATR-FTIR, ^{1}H-NMR, Morphology, TGA, DMA, and Mechanical Property

The attenuated total reflectance Fourier transform infrared (ATR-FTIR) spectra of membranes were recorded in the range of 4000 to 400 cm^{-1} by using a Nicolet-5700 spectrometer (Thermo Nicolet Co., USA). The ^{1}H-NMR spectra of membranes were obtained on a 400 MHz Bruker Avance III spectrometer (Bruker Co., Switzerland) using tetramethylsilane (TMS) and deuterated dimethyl sulfoxide (DMSO-d_6, δ = 2.50 ppm) as the internal standard and solvent, respectively. The morphologies and elemental information of the surface and cross-section of membranes were examined by using a multimode atomic force microscope (AFM) (Veeco Co., USA) in the tapping-mode and a scanning electron microscope (SEM) equipped with an energy dispersive spectroscopy (EDS) system (Nova Nano-230, FEI Co., USA). The cross-section of membranes was obtained using the freeze-fracture technique in liquid nitrogen. The thermogravimetric analysis (TGA) of membranes were carried out on a thermogravimetric analyzer (Q600, TA instrument Co., USA) with a heating rate of 10 °C min^{-1} from 35 to 800 °C under an argon atmosphere.

Dynamic mechanical analysis (DMA) of membranes was performed in tensile mode by using a dynamic mechanic analyzer (Q800, TA instrument Co., USA) with a constant frequency of 1 Hz and heating rate of 2 °C min^{-1} under a nitrogen atmosphere. The mechanical properties of dry membranes (15.0 mm × 3.0 mm) were studied on an Electromechanical Universal Testing Machine (SEMtester 100, Milliren Technologies Inc, USA) at room temperature with a cross-head speed of 3.0 mm min^{-1} of the specimen length.

2.2. Water Uptake, Swelling Ratio, and Contact Angle Measurements

The water uptake (*WU*) and through- and in-plane swelling ratios ($SR_{\Delta t}$ and $SR_{\Delta l}$) of membranes were determined according to the following procedures [1]. The membrane was dried at 50 °C for 24 h, and the weight, thickness, and length of membrane were accurately obtained. Afterwards, the membrane was soaked in DI water at 20 and 40 °C, respectively, for 24 h. After the membrane was taken out from the DI water, the excess water on the membrane surface was removed immediately. Then, the weight, thickness and length of membrane were immediately measured again. The *WU*, $SR_{\Delta t}$, and $SR_{\Delta l}$ of membrane were calculated by Equations (S1–S3),

$$WU = \frac{m_{\text{wet}} - m_{\text{dry}}}{m_{\text{dry}}} \times 100\% \quad \text{(S1)}$$

$$SR_{\Delta l} = \frac{l_{\text{wet}} - l_{\text{dry}}}{l_{\text{dry}}} \times 100\% \quad \text{(S2)}$$

$$SR_{\Delta t} = \frac{T_{\text{wet}} - T_{\text{dry}}}{T_{\text{dry}}} \times 100\% \quad \text{(S3)}$$

where m_{dry}, l_{dry}, and T_{dry} are the weight (g), length (mm), and thickness (mm) of the dried membrane, and m_{wet}, l_{wet}, and T_{wet} are the weight (g), length (mm), and thickness (mm) of the membrane after being soaked in DI water for 24 h at 20 or 40 °C.

A series of static contact angles were measured on the membrane surface by the sessile-drop method (JC2000D1, Shanghai Zhongchen Digital Technic Apparatus Co., Ltd, China) [2]. DI water, 3.0 mol L^{-1} H_2SO_4 solution, and 1.5 mol L^{-1} VO^{2+} + 3.0 mol L^{-1} H_2SO_4 solution were used as the probes, respectively. The sample membrane was fixed on slide glass, and the volume of the droplet was about 3.0 μL. The contact angle is obtained after 3 s of the droplet contacting the membrane surface at room temperature.

2.3. Ion Exchange Capacity, Proton Conductivity, Vanadium Ion Permeability, and Proton Selectivity

The ion exchange capacity (*IEC*) of membrane was measured by an acid-base titration method [3]. The *IEC* value is defined as the numbers of $-SO_3H$ groups per gram of a dry membrane. The membrane was washed with DI water and dried at 50 °C for 24 h, and its weight was measured. The dry membrane was then immersed in 1.0 mol L^{-1} of NaCl solution for 24 h to fully exchange H^+ ions with Na^+ ions. Lastly, the resulting solution was titrated with 0.03 mol L^{-1} NaOH solution using phenolphthalein as the indicator. The *IEC* of membrane was calculated by Equation (S4) as follows,

$$IEC = \frac{C_{\text{NaOH}} \times V_{\text{NaOH}}}{m_{\text{dry}}} \quad \text{(S4)}$$

where C_{NaOH} and V_{NaOH} are the concentration (mol L^{-1}) and volume (mL) of the consumed NaOH solution during the titration, respectively.

The proton conductivity (σ) of membrane was measured according to a reported method [4]. The membrane was immersed in 1.5 mol L^{-1} $VOSO_4$ + 3.0 mol L^{-1} H_2SO_4 solu-

tion for 24 h before test. The measurement was performed with a conductivity cell, which consists of two compartments separated by the membrane. Both compartments were filled with 1.5 mol L^{-1} $VOSO_4$ + 3.0 mol L^{-1} H_2SO_4 solutions. The effective area on each side of the membrane was 1.0 cm^2. The electric resistances of conductivity cell with membrane (R_1) and without membrane (R_2) were measured by electrochemical impedance spectroscopy (EIS) over a frequency range from 0.01 Hz to 100.0 kHz with an amplitude of 5.0 mV at room temperature using a CHI660b electrochemical workstation (Shanghai Chenhua Instruments Co., China). The σ of membrane was calculated by Equation (S5),

$$\sigma = \frac{T}{(R_1 - R_0) \times A} \tag{S5}$$

where the R_1 and R_0 are the area resistances of conductivity cell with and without the membrane, respectively, A is the effective area (cm^2) on each side of the membrane, and T is the thickness (cm) of the membrane.

The vanadium ion permeability (P) of membrane was evaluated using a membrane separated diffusion cell that was filled with 32.0 mL of 1.0 mol L^{-1} VO^{2+} + 2.0 mol L^{-1} H_2SO_4 solution in the left half-cell and 32.0 mL of 1.0 mol L^{-1} $MgSO_4$ + 2.0 mol L^{-1} H_2SO_4 solution in the right half-cell [1]. Besides, the effective area on each side of the sample membrane was 3.14 cm^2. The illustration of test setup is presented in Scheme S1. The VO^{2+} ions crossover through the membrane was evaluated by collecting about 3.0 mL of the sample solution from the right half-cell at a predetermined time interval of 720 min and analyzing it by using a UV-vis spectrometer (UV-1780, Shimadzu Co., Japan). The sample solution was transferred back to the right half-cell after the UV-vis test. Besides, the proton selectivity (PS) was defined as the ratio of proton conductivity to vanadium ion permeability to evaluate the comprehensive performance of the membrane. The P and PS of membrane were calculated by Equations (S6) and (S7),

$$V_\mathrm{R} \frac{\mathrm{d}C_\mathrm{R}(t)}{\mathrm{d}t} = A\frac{P}{d}(C_\mathrm{L} - C_\mathrm{R}(t)) \tag{S6}$$

$$PS = \frac{\sigma}{P} \tag{S7}$$

where C_R(t) is the concentration of vanadium ion (mol L^{-1}) in the right half-cell at a certain time, C_L is the initial concentration of vanadium ion (mol L^{-1}) in the left half-cell, V_R is the volume (cm^3) of the solution in the right half-cell, A is the effective area (cm^2) on each side of the membrane, d is the thickness (cm) of the membrane, and t is the time (min). Since the change of C_L is very small and C_L is much larger than C_R(t) during the permeability test, the vanadium ion concentration difference between the left and right half-cells could be treated as a constant and approximately equals to C_L (i.e., $C_L - C_R(t) \approx C_L$) for the calculation of P.

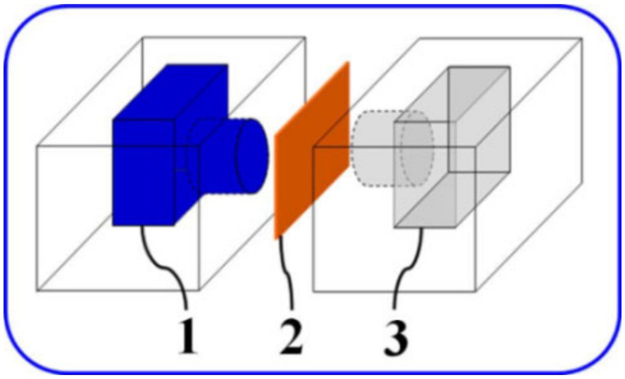

Scheme S1. The measurement device of vanadium ion permeability. (1) The left half-cell filled with 1.0 mol L^{-1} $VOSO_4$ + 2.0 mol L^{-1} H_2SO_4; (2) the sample membrane; (3) the right half-cell filled with 1.0 mol L^{-1} $MgSO_4$ + 2.0 mol L^{-1} H_2SO_4.

2.4. Ex−Situ Chemical Stability

The chemical stability of membrane was evaluated according to two kinds of different ex−situ methods [1]. The sample membrane (3.0 cm × 1.0 cm) was soaked in 22.0 mL 0.1 mol L^{-1} VO_2^+ + 3.0 mol L^{-1} H_2SO_4 solution at 40 °C and 22.0 mL 1.5 mol L^{-1} VO_2^+ + 3.0 mol L^{-1} H_2SO_4 solution at room temperature in sealed glass vials, respectively. The VO^{2+} ion concentration was recorded as an indicator to measure the degree of oxidation of the membrane. During the test, about 3.0 mL solution was taken from the vial on the 4th , 8th, 12th, 16th, and 20th day and analyzed for the VO^{2+} ion concentration by using an UV-vis spectrometer. The sample solution was transferred back to the original vial after the UV-vis test.

2.5. VRFB Single Cell Test

A schematic illustration of VRFB single cell is shown in Scheme S2. The VRFB single cell was fabricated by sandwiching the membrane between two pieces of 0.5 cm thick acid-activated graphite felt with an active area on each side of 30.0 cm^2, which served as the electrodes. The current collectors of VRFB single cell are two pieces of graphite bipolar plates (Liaoning Jingu Carbon Material Co., Ltd. China). The solutions of 60.0 mL of 1.7 mol L^{-1} $VO^{3.5+}$ + 4.6 mol L^{-1} H_2SO_4 (V^{3+}:VO^{2+} = 1:1) were served as the positive and negative electrolytes, respectively, and were cyclically pumped into the corresponding half-cells by magnetic pumps (MP-10RN, Shanghai Xinxishan Industrial Co., Ltd. China). To avoid the corrosion of graphite felt electrodes and graphite bipolar plates, the cut-off voltages for charge and discharge were set as 1.7 and 0.7 V, respectively. The charge-discharge of VRFB single cell was conducted by a battery testing system (CT2001B, Wuhan Land Co., Ltd, China). The coulombic efficiency (*CE*), energy efficiency (*EE*), and voltage efficiency (*VE*) of VRFB were calculated by Equations (S8–S10),

$$CE = \frac{C_{\text{dis}}}{C_{\text{ch}}} \times 100\% \quad \text{(S8)}$$

$$EE = \frac{E_{\text{dis}}}{E_{\text{ch}}} \times 100\% \quad \text{(S9)}$$

$$VE = \frac{EE}{CE} \times 100\% \quad \text{(S10)}$$

where C_{dis} and C_{ch} are the discharge and charge capacities (A h), and E_{dis} and E_{ch} are the discharge and charge energies (W h) in the process of charge-discharge, respectively.

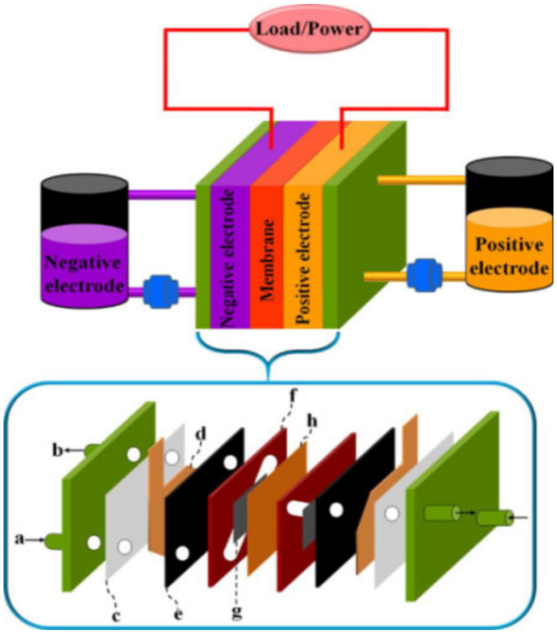

Scheme S2. Schematic illustration of a VRFB single cell. (a) liquid inlet; (b) liquid outlet; (c) gasket; (d) copper foil; (e) graphite bipolar plate; (f) plastic frame; (g) carbon felt; and (h) membrane. The plastic frame is used to hold the carbon felt and is basically in the same plane as the carbon felt.

3. Comparisons of the Physico-Chemical Properties and VRFB Performance of Pure SPI and SPI/Nafion Blend Membranes

Table S1. The physico-chemical properties and VRFB performance of pure SPI and SPI/Nafion blend membranes.

Membrane	SPI/Nafion Blend Membrane	Pure SPI Membrane
Water uptake (%) 20/40 °C	16.92/19.31	17.78/22.22
In-plane swelling ratio (%) 20/40 °C	2.57/3.87	2.87/4.17
Through-plane swelling ratio (%) 20/40 °C	13.52/17.24	14.51/17.72
DI water contact angle (°)	87.8	83.5
Tensile strength (MPa)	68.62	50.97
Young's modulus (GPa)	1.09	0.81
Elongations at break (%)	100.00	58.01
Ion exchange capacity (meq g^{-1})	1.61	1.50
Proton conductivity ($\times 10^{-2}$ S cm^{-1})	2.05	2.28
Vanadium ion permeability ($\times 10^{-7}$ cm^2 min^{-1}) 20/40 °C	1.25/2.23	0.74/1.29
Proton selectivity ($\times 10^5$ S min cm^{-3})	1.64	3.08
Ex–situ chemical stability		
0.1 M VO_2^+ + 3.0 M H_2SO_4 at 40 °C	0.00572	0.00658
1.5 M VO_2^+ + 3.0 M H_2SO_4 at room temperature	0.02047	0.02272
$C_{VO^{2+}}$ after 20 days (mol L^{-1})		
Coulombic efficiencies (%) (60 mA cm^{-2})	96.0	99.6
Voltage efficiencies (%) (60 mA cm^{-2})	80.7	77.3
Energy efficiencies (%) (60 mA cm^{-2})	77.5	77.0

4. Comparisons of the Morphologies, Physico-Chemical Properties, and Battery Performance of VRFBs Assembled with SPI/Nafion and Nafion 115 Membranes

Table S2. Comparisons of the morphologies, physico-chemical properties, and battery performance of VRFBs assembled with SPI/Nafion and Nafion 115 membranes. The properties of SPI/Nafion membrane superior to those of Nafion 115 membrane are marked in red.

Membrane	SPI/Nafion Blend Membrane	Commercial Nafion 115
Surface roughness parameter (nm)	0.418	1.83
Surface area difference (%)	0.110	0.965
Weight retention at 800 °C (%)	50.53	0.11
Storage modulus (MPa)	810.6 (at 400 °C)	0 (at 242 °C)
Glass transition temperature (°C)	329	121
Water uptake (%) 20/40 °C	16.92/19.31	15.28/17.68
In-plane swelling ratio (%) 20/40 °C	2.57/3.87	2.35/3.49
Through-plane swelling ratio (%) 20/40 °C	13.52/17.24	11.96/16.10
DI water contact angle (°)	87.8	101.5
3.0 M H_2SO_4 contact angle (°)	91.3	104.2
1.5 M VO^{2+} + 3.0 M H_2SO_4 contact angle (°)	89.9	102.1
Tensile strength (MPa)	68.62	12.79
Young's modulus (GPa)	1.09	0.06
Elongations at break (%)	100.00	223.17
Ion exchange capacity (meq g^{-1})	1.61	0.74
Proton conductivity ($\times 10^{-2}$ S cm^{-1})	2.05	6.00
Vanadium ion permeability ($\times 10^{-7}$ cm^2 min^{-1}) 20/40 °C	1.25/2.23	13.59/35.97
Proton selectivity ($\times 10^{5}$ S min cm^{-3})	1.64	0.44
Ex−situ chemical stability		
0.1 M VO_2^{+} + 3.0 M H_2SO_4 at 40 °C	0.00572	0.00215
1.5 M VO_2^{+} + 3.0 M H_2SO_4 at room temperature	0.02047	0.01798
$C_{VO^{2+}}$ after 20 days (mol L^{-1})		
Coulombic efficiencies (%) (200 - 20 mA cm^{-2})	97.77–93.88	95.59–92.53
Voltage efficiencies (%) (200 - 160 mA cm^{-2})	58.46–61.37	60.04–61.78
Voltage efficiencies (%) (140 - 20 mA cm^{-2})	65.07–89.96	64.31–89.23
Energy efficiency (%) (200 mA cm^{-2})	57.16	57.39
Energy efficiencies (%) (180 - 20 mA cm^{-2})	58.10–84.45	57.76–82.56

5. Comparison of the Ex−Situ Chemical Stability of SPI/Nafion Blend Membrane and Other SPI-Based Membranes

Table S3. Comparison of the ex situ chemical stability of SPI/Nafion blend membrane and other SPI-based membranes.

Membrane	Test condition	Immersing time (day)	$C_{VO^{2+}}$ (mol L^{-1})	Reference
Semi-fluorinated sulfonated polyimide membrane	0.1 mol L^{-1} VO_2^{+} + 3.0 mol L^{-1} H_2SO_4 at 40 °C	22	0.04351	[5]
Based-APABI sulfonated polyimide membrane	0.1 mol L^{-1} VO_2^{+} + 3.0 mol L^{-1} H_2SO_4 at 40 °C	12	0.02403	[6]
Based-BAPP sulfonated polyimide membrane			0.03035	

Based-MDA sulfonated polyimide membrane			0.03599	
Sulfonated poly(imide-siloxane) membrane	0.1 mol L^{-1} VO_2^+ + 3.0 mol L^{-1} H_2SO_4 at 25 °C	12	about 0.0125	[7]
Based-ODA sulfonated polyimide membrane	0.1 mol L^{-1} VO_2^+ + 3.0 mol L^{-1} H_2SO_4 at 40 °C	20	0.01131	[4]
Branched side-chain-type sulfonated polyimide membrane			0.00625	
Based-APABI branched sulfonated polyimide	0.1 mol L^{-1} VO_2^+ + 3.0 mol L^{-1} H_2SO_4 at 40 °C	16	about 0.014	[8]
Based-BAPP branched sulfonated polyimide			about 0.009	
Based-MDA branched sulfonated polyimide			about 0.012	
Side-chain-type fluorinated sulfonated polyimide membrane	0.1 mol L^{-1} VO_2^+ + 3.0 mol L^{-1} H_2SO_4 at 40 °C	20	0.00658	[1]
	1.5 mol L^{-1} VO_2^+ + 3.0 mol L^{-1} H_2SO_4 at room temperature		0.02272	
Sulfonated polyimide/polyvinyl alcohol blend membrane	0.15 mol L^{-1} VO_2^+ + 3.0 mol L^{-1} H_2SO_4	about 180 h	about 0.08	[9]
c-FbSPI-60 ionic cross-linking membrane	0.1 mol L^{-1} VO_2^+ + 3.0 mol L^{-1} H_2SO_4 at 40 °C	16	about 0.032	[10]
bSPI/MoS_2-ns composite membranes	0.1 mol L^{-1} VO_2^+ + 3.0 mol L^{-1} H_2SO_4 at 40 °C	14	about 0.0062	[11]
bSPI/SiC(PDMS)-1.5% composite membranes	0.1 mol L^{-1} VO_2^+ + 3.0 mol L^{-1} H_2SO_4 at 40 °C	30	about 0.00712	[12]
SPI5/PVA5 membranes	0.15 mol L^{-1} VO_2^+ + 3.0 mol L^{-1} H_2SO_4	28	about 0.00275	[13]
Sulfonated polyimide/Nafion blend membrane	0.1 mol L^{-1} VO_2^+ + 3.0 mol L^{-1} H_2SO_4 at 40 °C	20	0.00572	this work
	1.5 mol L^{-1} VO_2^+ + 3.0 mol L^{-1} H_2SO_4 at room temperature		0.02047	

6. Comparisons of the Morphology Parameters, Physico-Chemical Properties of SPI/Nafion Blend Membrane after 400-time VRFB Cycling Test and Nafion 115 Membrane

Table S4. Comparisons of the morphology parameters, thermal stability, and rheological properties between SPI/Nafion after 400-time VRFB cycling test and fresh Nafion 115 membranes. The properties of SPI/Nafion blend membrane after 400-time VRFB cycling test superior to those of Nafion 115 membrane are marked in red.

Membrane	SPI/Nafion blend membrane after 400-time VRFB cycling test	Commercial Nafion 115
Surface roughness parameter (nm)	1.60 (facing the positive electrode)<br>0.950 (facing the negative electrode)	1.83
Surface area difference (%)	0.807 (facing the positive electrode)<br>0.764 (facing the negative electrode)	0.965
Weight retention at 800 °C (%)	44.93	0.11
Storage modulus (MPa)	914.5<br>(at 400 °C)	0<br>(at 242 °C)
Glass transition temperature (°C)	320	121

7. The Molecular Structures of SPI and Nafion Polymers

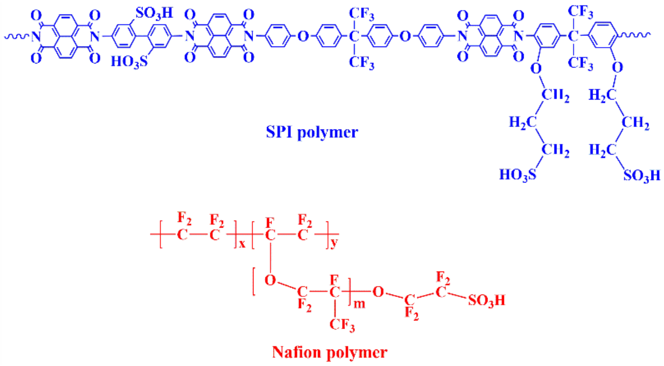

Figure S1. The molecular structures of SPI and Nafion polymers.

8. ATR-FTIR Study of SPI/Nafion Blend Membranes Fabricated Using Different Solvents

The ATR-FTIR spectra of SPI/Nafion blend membranes fabricated using *m*-cresol, DMSO, NMP, DMF, and DMAc as the membrane-casting solvents were obtained and shown in Figure S2.

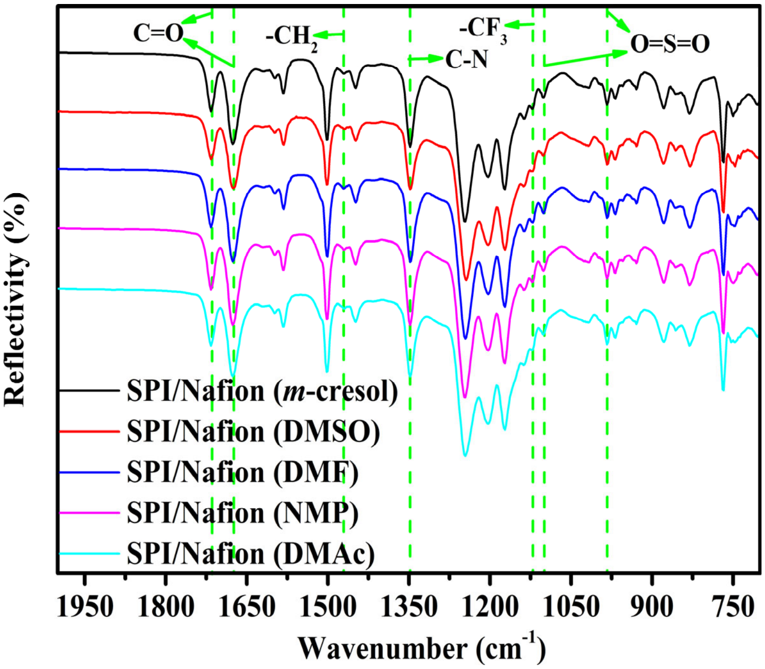

Figure S2. The ATR-FTIR spectra of SPI/Nafion blend membranes fabricated using *m*-cresol, DMSO, DMF, NMP, and DMAc as the membrane-casting solvents, respectively.

9. ^{1}H-NMR study of SPI/Nafion blend membranes fabricated using different solvents

The ^{1}H-NMR spectra of SPI/Nafion blend membranes fabricated using *m*-cresol, DMSO, NMP, DMF, and DMAc as the membrane-casting solvents are presented in Figure S3.

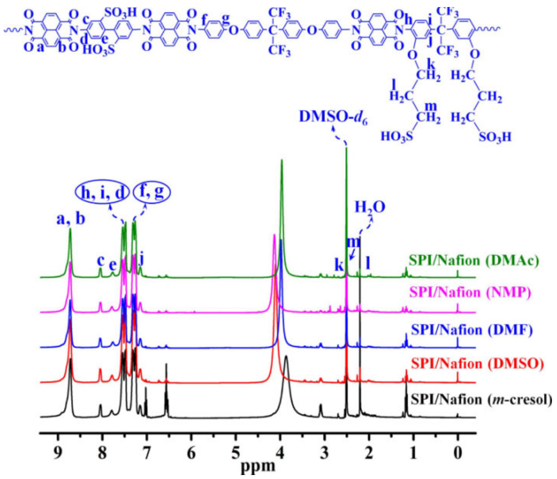

Figure S3. The ^{1}H-NMR spectra of SPI/Nafion blend membranes fabricated using *m*-cresol, DMSO, DMF, NMP, and DMAc as the membrane-casting solvents, respectively.10. TGA curves of pure SPI, Nafion 115, and SPI/Nafion blend membranes fabricated using different solvents.

The TGA curves of SPI/Nafion blend membranes fabricated using different membrane-casting solvents (i.e., *m*-cresol, DMSO, DMF, NMP, or DMAc), Nafion 115, and pure SPI membranes are presented in Figure S4.

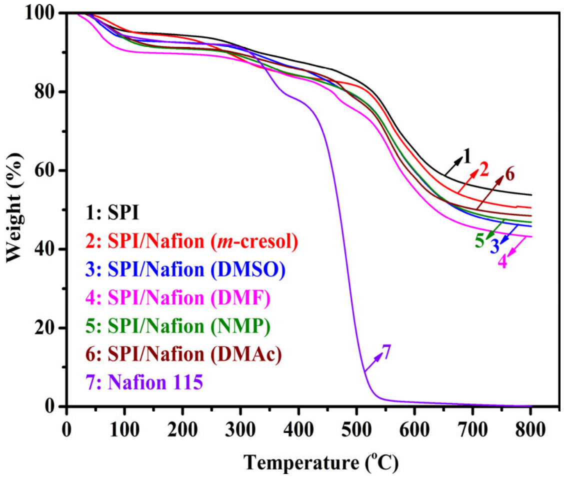

Figure S4. TGA curves of the pure SPI membrane, the Nafion 115 membrane, and the SPI/Nafion blend membranes fabricated using different solvents including *m*-cresol, DMSO, DMF, NMP, and DMAc.

11. The contact angles of SPI/Nafion and Nafion 115 membranes

The contact angles of SPI/Nafion and Nafion 115 membranes by using DI water, 3.0 mol L^{-1} H_2SO_4 solution, and 1.5 mol L^{-1} VO^{2+} + 3.0 mol L^{-1} H_2SO_4 solution (i.e., a VRFB electrolyte) as probes are shown in Figure S5.

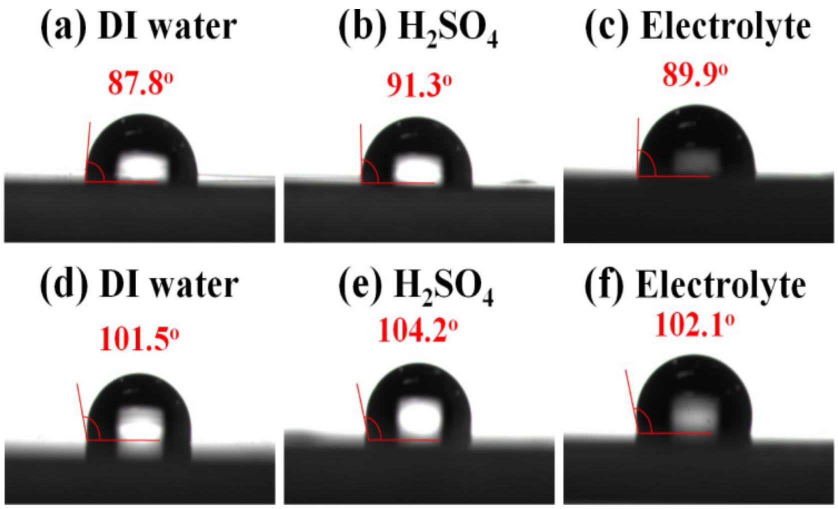

Figure S5. The contact angles of (a-c) SPI/Nafion and (d-f) Nafion 115 membranes. The concentration of H_2SO_4 is 3.0 mol L^{-1}. The electrolyte consists of 1.5 mol L^{-1} VO^{2+} and 3.0 mol L^{-1} H_2SO_4.

12. Battery Performances of SPI/Nafion and Nafion 115 Membranes

The *CE*s, *VE*s, and *EE*s are obtained by calculating the average values of the efficiencies of the 30 cycles at each current density, and the results are illustrated in Figure S6.

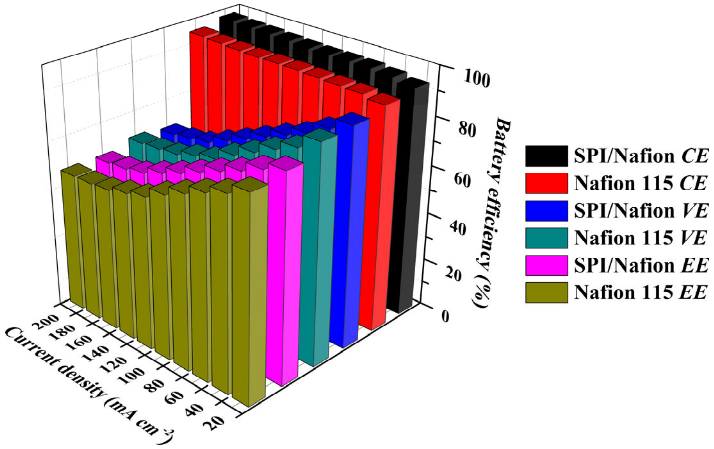

Figure S6. The coulombic efficiencies (*CE*s), energy efficiencies (*EE*s), and voltage efficiencies (*VE*s) of SPI/Nafion and Nafion 115 membranes under current densities from 200 to 20 mA cm^{-2}.

13. The Discharge Capacity Retentions of SPI/Nafion and Nafion 115 Membranes

The discharge capacity retention results of SPI/Nafion blend membrane and Nafion 115 membrane at another 100-time cycling VRFB tests at 100 mA cm^{-2} after the first 300-time cycling test are shown in Figure S7.

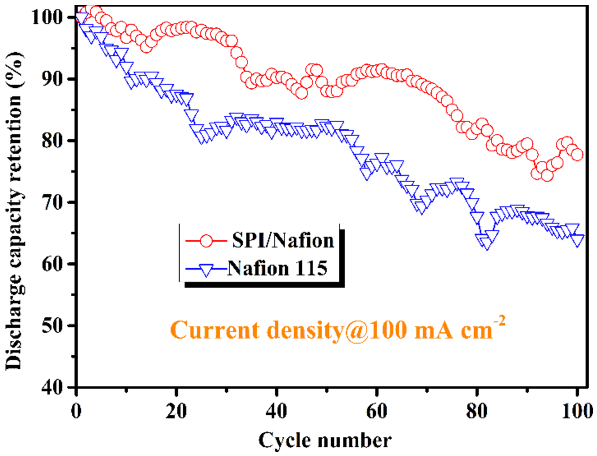

Figure S7. The discharge capacity retentions of SPI/Nafion and Nafion 115 membranes at 100 mA cm^{-2}.

14. 500-Time Cycling Charge-Discharge Performance of SPI/Nafion Blend Membrane

The 500-time VRFB charge-discharge cycling test was performed at 100 mA cm^{-2} and the results are presented in Figure S8. The *CE* and *EE* of SPI/Nafion blend membrane show no obvious decrease over 500 cycles, demonstrating the excellent cycling performance and chemical stability of SPI/Nafion blend membrane.

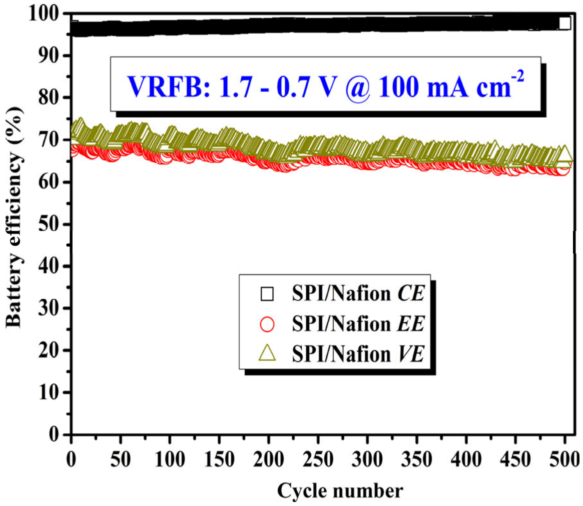

Figure S8. The 500-time cycling performance of SPI/Nafion blend membrane at 100 mA cm^{-2}.

15. TGA Curves of Membranes

The TGA curves of SPI/Nafion blend membrane before and after 400-time VRFB cycling test are shown in Figure S9.

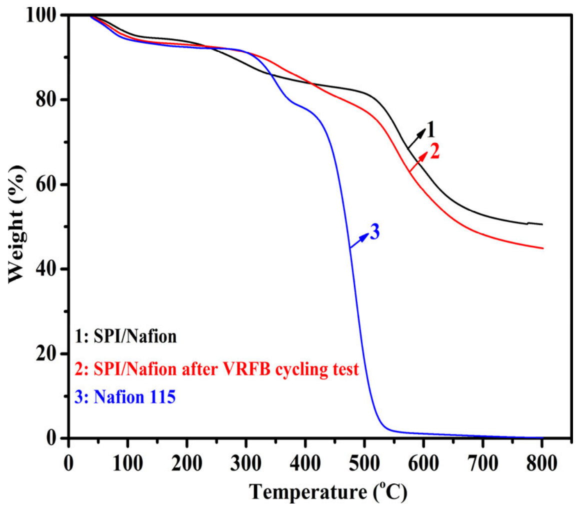

Figure S9. TGA curves of SPI/Nafion membrane before and after 400-time VRFB cycling test.Supporting References.

References

1. Li, J.C.; Yuan, X.D.; Liu, S.Q.; He, Z.; Zhou, Z. Li, A.Q. A low-cost and high-performance sulfonated polyimide proton-conductive membrane for vanadium redox flow/static batteries. ACS Appl. Mater. Interfaces **2017**, *9*, 32643-32651.
2. Lee, C.H.; Lee, S.Y.; Lee, Y.M.; Lee, S.Y.; Rhim, J.W.; Lane, O.; McGrath, J.E. Surface-fluorinated proton-exchange membrane with high electrochemical durability for direct methanol fuel cells. ACS Appl. Mater. Interfaces **2009**, *1*, 1113-1121.
3. Ji, Y.; Tay, Z.Y.; Li, S.F.Y. Highly selective sulfonated poly(ether ether ketone)/titanium oxide composite membranes for vanadium redox flow batteries. J. Membr. Sci. **2017**, *539*, 197-205.
4. Li, J.C.; Liu, S.Q.; He, Z.; Zhou, Z. A novel branched side-chain-type sulfonated polyimide membrane with flexible sulfoalkyl pendants and trifluoromethyl groups for vanadium redox flow batteries. J. Power Sources **2017**, *347*, 114-126.
5. Li, J.C.; Liu, S.Q.; He, Z.; Zhou, Z. Semi-fluorinated sulfonated polyimide membranes with enhanced proton selectivity and stability for vanadium redox flow batteries. Electrochim. Acta **2016**, *216*, 320-331.
6. Zhang, Y.P.; Li, J.C.; Zhang, H.; Zhang, S.; Huang, X.D. Sulfonated polyimide membranes with different non-sulfonated diamines for vanadium redox battery applications. Electrochim. Acta **2014**, *150*, 114-122.
7. Zhang, S.; Li, J.C.; Huang, X.D.; Zhang, Y.P.; Zhang, Y.D. Sulfonated poly(imide-siloxane) membrane as a low vanadium ion permeable separator for a vanadium redox flow battery. Polym. J. **2015**, *15*, 1-8.
8. Pu, Y.; Huang, X.D.; Yang, P. Zhou, Y.Q. Xuan, S.S.; Zhang, Y.P. Effect of non-sulfonated diamine monomer on branched sulfonated polyimide membrane for vanadium redox flow battery application. Electrochim. Acta **2017**, *241*, 50-62.
9. Liu, S.; Wang, L.H.; Zhang, B.; Liu, B.Q.; Wang, J.J.; Song, Y.L. Novel sulfonated polyimide/polyvinyl alcohol blend membranes for vanadium redox flow battery applications. J. Mater. Chem. A **2015**, *3*, 2072-2081.
10. Yang, P.; Xuan, S.S.; Wang, Y.L.; Zhang, Y.P.; Li, J.C.; Zhang, H.P. Branched sulfonated polyimide membrane with ionic cross-linking for vanadium redox flow battery application. J. Power Sources **2019**, *438*, 226993.
11. Pu, Y.; Zhu, S.; Wang, P.H.; Zhou, Y.Q.; Yang, P.; Xuan, S.S.; Zhang, Y.P.; Zhang, H.P. Novel branched sulfonated polyimide/molybdenum disulfide nanosheets composite membrane for vanadium redox flow battery application. Appl. Surf. Sci. **2018**, *448*, 186-202.
12. Zhang, Y.P.; Pu, Y.; Yang, P.; Yang, H.Y.; Xuan, S.S.; Long, J.; Wang, Y.L.; Zhang, H.P. Branched sulfonated polyimide/functionalized silicon carbide composite membranes with improved chemical stabilities and proton selectivities for vanadium redox flow battery application. J. Mater. Sci. **2018**, *53*, 14506-14524.
13. Yu, H.L.; Xia, Y.F.; Zhang, H.W.; Wang, Y.H. Preparation of sulfonated polyimide/polyvinyl alcohol composite membrane for vanadium redox flow battery applications. Polym. Bull. **2021**, *78*, 4183-4204.
